# A membrane-free flow electrolyzer operating at high current density using earth-abundant catalysts for water splitting

**DOI:** 10.1038/s41467-021-24284-5

**Published:** 2021-07-06

**Authors:** Xiaoyu Yan, Jasper Biemolt, Kai Zhao, Yang Zhao, Xiaojuan Cao, Ying Yang, Xiaoyu Wu, Gadi Rothenberg, Ning Yan

**Affiliations:** 1 School of Physics and Technology, Wuhan University, Wuhan, China; 2 Suzhou Institute of Wuhan University, Suzhou, China; 3 Van’t Hoff Institute for Molecular Sciences (HIMS), University of Amsterdam, Amsterdam, The Netherlands; 4 Hubei Key Laboratory of Theory and Application of Advanced Materials Mechanics, Wuhan University of Technology, Wuhan, China

**Keywords:** Electrocatalysis, Renewable energy, Electrocatalysis, Nanoscale materials

## Abstract

Electrochemical water splitting is one of the most sustainable approaches for generating hydrogen. Because of the inherent constraints associated with the architecture and materials, the conventional alkaline water electrolyzer and the emerging proton exchange membrane electrolyzer are suffering from low efficiency and high materials/operation costs, respectively. Herein, we design a membrane-free flow electrolyzer, featuring a sandwich-like architecture and a cyclic operation mode, for decoupled overall water splitting. Comprised of two physically-separated compartments with flowing H_2_-rich catholyte and O_2_-rich anolyte, the cell delivers H_2_ with a purity >99.1%. Its low internal ohmic resistance, highly active yet affordable bifunctional catalysts and efficient mass transport enable the water splitting at current density of 750 mA cm^−2^ biased at 2.1 V. The eletrolyzer works equally well both in deionized water and in regular tap water. This work demonstrates the opportunity of combining the advantages of different electrolyzer concepts for water splitting via cell architecture and materials design, opening pathways for sustainable hydrogen generation.

## Abstract (web summary)

Seawater electrolysis is promising for grid-scale H_2_ production without freshwater reliance, but high energy costs and detrimental Cl chemistry reduce its practical potential. Here, authors developed an energy-saving hybrid seawater electrolyzer for chlorine-free H_2_ production and N_2_H_4_ degradation.

## Introduction

Replacing the fossil fuels in today’s energy portfolio is a grand challenge of the sustainable society^1–3^. Among all the proposed alternatives, hydrogen generated electrochemically using water and renewable energy sources has been regarded as one of the best, if not the ultimate, solutions^4–7^. The conventional alkaline water electrolyzer dates back in 1789^8,9^. It enables the use of cost-effective catalysts for both the hydrogen evolution reaction (HER) and oxygen evolution reaction (OER), enjoying low capital expenditure and operating expenses^10^. Yet, as the anode and cathode are separated by a porous diaphragm, the potential mixture of H_2_ and O_2_ brings safety concerns. The resulting high ohmic resistance often precludes the operation at high current density^11–13^. Recently, the polymer membrane electrolyzer has been highlighted as the next-generation candidate^14^. Particularly, the proton-exchange membrane (PEM) based one dominates, allowing operation at high current density (>1 A cm^−^^2^) while delivering pressurized high-purity H_2_ ( > 99%)^15,16^. Nonetheless, only noble metal catalysts can survive in the corrosive environment of the cell^17^. Besides, deionized water must be used to reduce membrane degradation^18,19^. The high cost is thus a prominent barrier impeding large-scale application^20,21^. Anion-exchange membrane (AEM) electrolyzers seem preferable in this context, as affordable materials can be used. The problem is that the durability of state-of-the-art AEMs is often only ~1000 h^22^.

Since the performance of conventional water electrolyzers is inherently constrained by their architecture and configuration, various cell designs have emerged to tackle these limitations. One promising alternative is to circumvent the need for a membrane by decoupling the OER and HER spatially and/or temporarily in overall water splitting^17,23–26^. Compared with the electrolyzers containing solid membranes, this membrane-free cell is less complex, potentially more robust in the harsh reaction environments, and cheaper. Moreover, it can operate using different aqueous electrolyte^27^. For example, Cronin and co-workers used phosphomolybdic acid as the “electron buffer” to spatially decouple the OER and HER. Other (in)soluble redox mediators were also proposed, such as [Fe(CN)_6_]^4−^ /[Fe(CN)_6_]^3− ^, V^3+^/V^4+^ and NiOOH/Ni(OH)_2_^28–32^. A further advantage of this approach is that it offers the opportunity of coupling water splitting with battery technologies for concurrent energy storage^33,34^. Membrane-free electrolysis is scalable. For instance, the divergent electrode-flow-through (DEFT) membraneless alkaline electrolysis takes advantage of the divergent flow of electrolyte to maintain gas separation while delivering excellent performance in a pilot plant^35,36^.

Notwithstanding these advantages, many membrane-free cells suffer from large ohmic drops. In fact, while the ionic conductivity of concentrated KOH and H_2_SO_4_ solution is higher than that of the membrane, yet the travel distance of ions is typically at the millimeters scale, which is much longer than the thickness of membrane electrolyte (≈100–200 μm)^37^. This is one of the key factors leading to the much lower working current densities and higher working voltages (>2.5 V)^38^. Herein, we design a membrane-free flow electrolyzer featuring a sandwich-like architecture for decoupled water splitting. Though comprising of two symmetric compartments, it features the low internal ohmic resistance and efficient mass transport, allowing the operation at high current density with low overpotential. Importantly, the bifunctional catalysts are based on affordable Fe/Co compounds, which opens opportunities for real-life applications.

## Results

### Membrane-free flow electrolyzer configuration

**Fig. 1:**
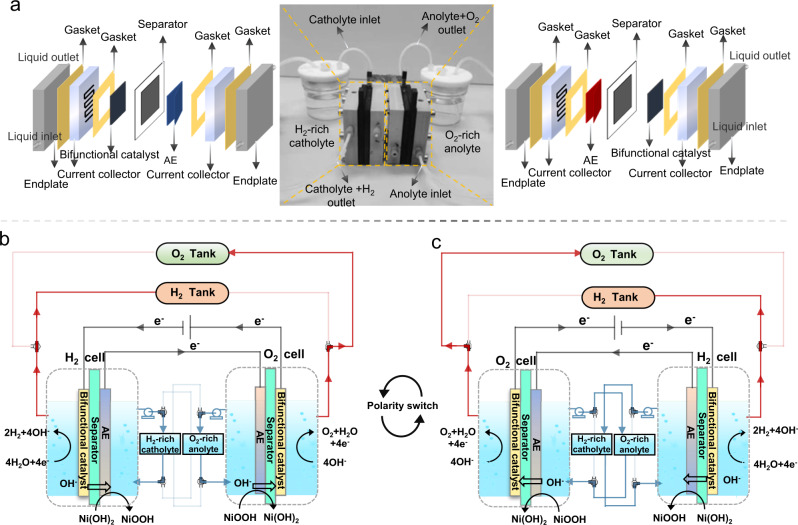
The configuration and working principle of the membrane-free flow electrolyzer. **a** The components and assembly of the membrane-free flow electrolyzer. **b**, **c** The detailed cyclical working principle of the membrane-free flow electrolyzer, showing how the roles of each compartment are reversed during the operation.

Figure 1a shows the assembly and working principle of the membrane-free flow electrolyzer (MFE). The cell comprises two physically separated compartments, connected by wires that allows the transfer of electrons, for oxygen evolution and hydrogen evolution reactions, respectively. Both compartments share the same sandwich-like configuration, comprising of endplates, current collectors, a bifunctionally active electrode, a 130 μm-thick porous separator, and an auxiliary electrode (AE, NiOOH/Ni(OH)_2_ redox couple). The anolyte (O_2_-rich 1.0 M KOH solution) and catholyte (H_2_-rich 1.0 M KOH solution) are directed to flow to the designated compartment continuously during the reaction. Thanks to this closely packed sandwich structure with a much shorter travel distance of the ions, the ohmic resistance of our membrane-free cell is comparable with that of the proton exchange membrane water electrolyzer (PEMEC, vide infra). Together with the separation of two reaction chamber, this opens opportunities for the cell to work at high current densities that are comparable with PEMEC.

Our cell works cyclically during water splitting. In the cycle when the left compartment is the cathode (see Fig. 1b), the bifunctional catalyst on the working electrode catalyzes the HER in the catholyte, generating OH^-^ anions which pass through the separator and react with Ni(OH)_2_ at the AE to form NiOOH. The evolved H_2_ gas is collected in the H_2_ tank via a 3-way valve. In the meantime, the working electrode in the other compartment catalyzes the OER in the anolyte, using the OH^−^ ions supplied by the conversion of NiOOH into Ni(OH)_2_. The formed O_2_ also flows through another 3-way valve to the O_2_ tank (or to the vent upon request). When the AE materials are nearly all converted, the continuous operation of the system is guaranteed by switching the electrical current polarity of the cell as shown in Fig. 1c and Supplementary Fig. 8. As the left compartment becomes the anode in the following cycle, the catholyte is replaced by the O_2_-rich anolyte beforehand. This swap was designed to avoid the mixture of the residual H_2_ and O_2_ left in the compartment. The 3-way valve is also switched to the direction to ensure the formed O_2_ flows to O_2_ tank. Meanwhile, the right-side compartment and the accessories behave accordingly to ensure the generation and collection of H_2_ with high purity. Because the electrode catalyst is OER/HER bifunctionally active, this cyclical operation shows essentially identical and excellent performance in all cycles.

### Bifunctional electrocatalyst

**Fig. 2:**
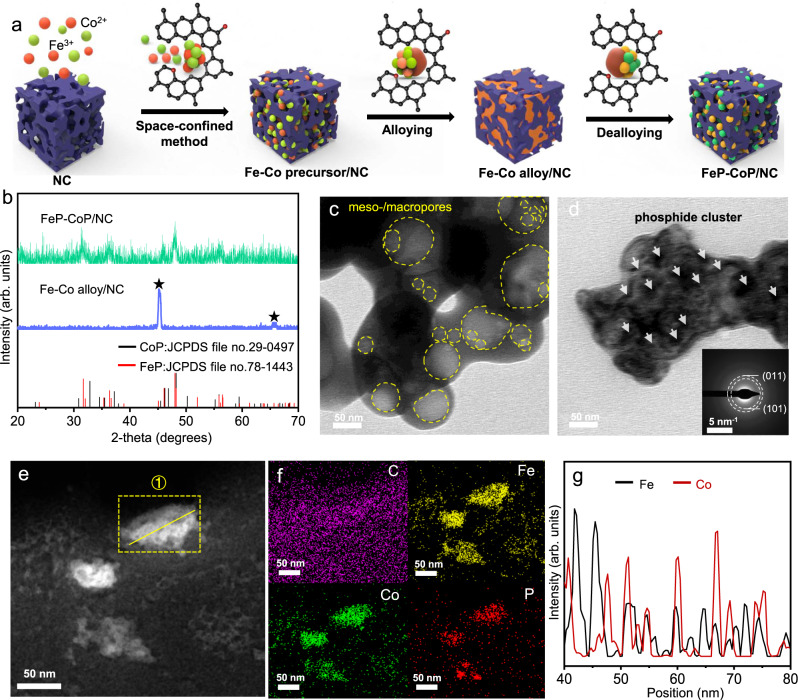
The schematic synthetic procedure and characterizations of FeP–CoP/NC bifunctional catalyst. **a** Representative illustration of the synthetic procedure of FeP–CoP/NC; **b** XRD patterns of Fe–Co alloy/NC and FeP–CoP/NC; TEM micrographs of **c** pristine NC with meso/macropores and **d** FeP-CoP/NC, the inset is the SAED pattern of FeP–CoP domains; **e** HAADF image of FeP–CoP/NC, EDX line scan position is indicated; **f** EDX elemental mappings of C, Fe, Co, and P; **g** EDX line scan of the nanoparticle in **e**.

To ensure the excellent bifunctional activity, efficient mass transport, high electrical conductivity, and good physical compatibility with the electrode, we designed the hierarchically porous catalyst comprising FeP, CoP and nitrogen doped carbon (NC). This hybrid system, denoted as FeP–CoP/NC, was synthesized through the sequential processes by confining Fe–Co precursor in the mesopores of NC by infiltration, forming Fe–Co bimetallic alloy by pyrolysis, and phosphidation giving the interconnected FeP and CoP quantum dots (see the schematic in Fig. 2a). Figure 2b shows the X-ray diffraction (XRD) pattern of the pyrolyzed NC embedded with the Co–Fe precursor. The reducing atmosphere facilitated the formation of Fe–Co bimetallic alloy. After phosphidation, the coexistence of FeP (PDF No. 78-1443) and CoP (PDF No. 29-0497) was confirmed^39–45^. The crystallized grain size of both FeP and CoP, calculated using the Scherrer equation, was ~3.4 nm. In fact, as FeP and CoP are immiscible, doping FeP with Co or vice versa to optimize the catalytic performance is challenging^46,47^. The alloying step created the homogenously dispersed Fe and Co phase at the atomic level, contributing to the generation of the small nanocrystals of CoP and FeP. Such small domains maximized the length of the FeP–CoP interface for improved bifunctional catalysis (vide infra)^48^.

The morphology of the pristine NC was characterized using transmission electron microscope (TEM, Fig. 2c). This hierarchically porous structure contains abundant micro-, meso-, and macropores (the latter two are demonstrated by the circles, also see the N_2_ adsorption isotherm and the corresponding pore size distribution analysis in Supplementary Fig. 10). After embedding FeP and CoP, the TEM image in Fig. 2d shows that the phosphide cluster are mainly confined in the large pores of NC, yet not fully occupied the mesopore space. The comparison of the pore size distribution curve also indicates the pores, sizing between 20 and 50 nm, were retained after filling in the phosphide catalyst. The carbon backbones minimized the ohmic loss, whereas the remaining pores is vital for the mass transport. Thus, this hybrid catalyst is critical for operating the electrolyzer at high current density.

The formation of FeP and CoP cluster was also verified by energy-dispersive X-ray spectroscopy (EDX) using high-angle annular dark field detector in the scanning transmission electron microscope mode (HAADF-STEM). The clusters were <50 nm in diameter in Fig. 2e. Albeit that CoP and FeP are immiscible, the elemental mappings show that both Fe and Co are uniformly distributed within the cluster (see Fig. 2f). The EDX line scan confirmed the domain size of both phosphides was 2–3 nm, in agreement with the XRD analysis. This conclusion was also supported by the selected area electron diffraction (SAED, see the inset of Fig. 2d). The small diffuse halo rings, stemming from the (011) and (101) plane of FeP and CoP, respectively, showed that both the CoP and FeP domains were nanocrystalline.

**Fig. 3:**
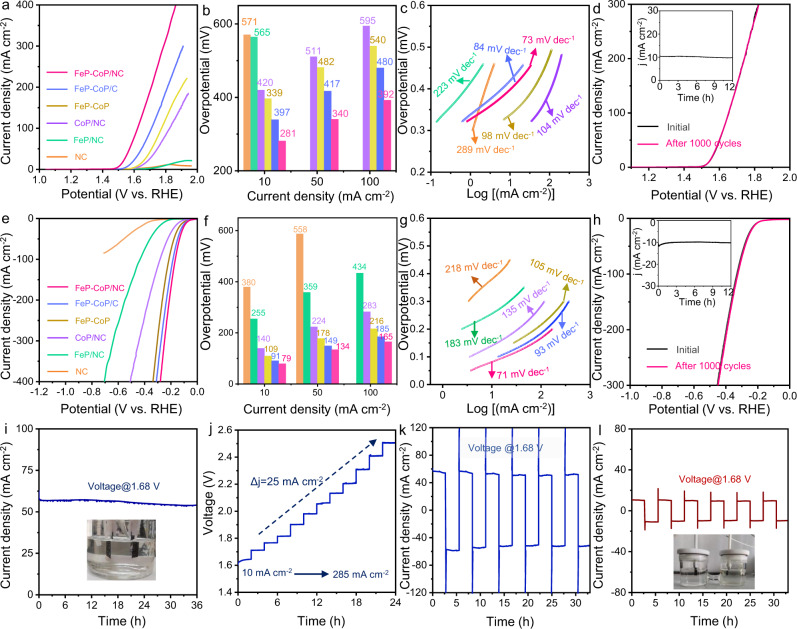
The electrocatalytic performance of FeP–CoP/NC. The comparisons of **a** LSVs, **b** overpotentials at various current densities, **c** Tafel slopes, and **d** stability with the controls in OER. The comparisons of **e** LSVs, **f** overpotentials at various current densities, **g** Tafel slopes, and **h** stability with the controls in HER. The electrolyte is 1.0 M KOH, the scan rate is 5 mV s^−1^, post-*i*R compensation is applied. The performance of overall water splitting using FeP–CoP/NC as the catalysts: **i** the *j*–*t* curve at 1.68 V; **j** the *V*–*t* curve at various current densities and **k** the *j*–*t* curve at ±1.68 V with the potential polarity swapped every 3.3 h in a single compartment cell; **l** the *j*–*t* curve at ±1.68 V with the potential polarity swapped every 3.3 h in a H-cell. No *i*R compensation was applied.

We first used the standard three-electrode setup in 1.0 M KOH to evaluate the OER activities of the prepared electrocatalysts on the glassy carbon working electrode. Figure 3a compares the linear sweep voltammograms (LSVs) of FeP–CoP/NC with the controls. Gaining the current densities of up to 10, 50, 100 mA cm^–2^, FeP–CoP/NC, with 1:1 molar ratio of Fe and Co, required the lowest overpotentials (*η*) of 281, 340, and 392 mV, respectively. In contrast, samples of hybrid phosphide on commercial carbon (FeP–CoP/C), FeP–CoP, and NC all showed much higher overpotentials and larger Tafel slopes (see Fig. 3c). In fact, the commercial carbon lacked the mesopores which suffered higher concentration overpotential at high current density. Unsupported FeP–CoP was much less conductive, thus experiencing significant ohmic loss. This was proven by the Nyquist plot from the electrochemical impedance spectroscopy (EIS, see Supplementary Fig. 11). Particularly, the molar ratio of Fe/Co (1:0, 1:0.5, 1:1, 0.5:1, and 0:1) was a critical factor affecting the OER activity (see Supplementary Fig. 12). This might be linked to the electronic structure and the synergistic effect of the catalyst (vide infra). We also studied the stability of FeP–CoP/NC via performing 1000 cyclic voltammetry (CV) cycles (see Fig. 3d). Negligible change of LSVs was observed after the cycles. Besides, we also biased the cell at the overpotential of 281 mV, steady OER current was recorded over the 12 h test (see the inset of Fig. 3d). The excellent bifunctionality of FeP–CoP/NC was then exhibited in the HER. At the benchmark 10 mA cm^–2^ current density, FeP–CoP/NC delivered an overpotential of 79 mV, comparable with the state-of-the-art catalysts (see Supplementary Tables 1 and 2). The Tafel slope was 71 mV dec^-1^, less steep than that of FeP–CoP/C (93 mV dec^−1^), FeP–CoP (105 mV dec^−1^), indicating more rapid reaction kinetics. Meanwhile, no activity decay was recorded after the stability test in Fig. 3h.

We then explored the bifunctionality, stability, and system compatibility of FeP–CoP/NC under practical conditions. No iR compensation was applied in all the water electrolysis studies below. The hybrid catalyst was printed onto the carbon cloth as both the anode and cathode of a single compartment electrolyzer for alkaline water splitting in 1.0 M KOH. At 1.68 V (*η* = 450 mV) in the overall water splitting reaction, the current density reached 55 mA cm^–2^, which was maintained for 36 h of electrolysis (Fig. 3i). In the same cell, we also exampled the stability and the dynamic response of the electrode chronopotentiometrically. Figure 3j shows the applied voltage responses of the cell when the current density was stepped up from 10 to 285 mA cm^–2^ (Δ*j* = 25 mA cm^–2^). The highest potential reached 2.51 V, due to the large ohmic resistance of the cell. Nevertheless, the potential was stabilized rapidly after each current ramp. The cyclic stability of the catalyst during cycled OER and HER was carried out by switching the potential polarity periodically (±1.68 V, 3.3 h per cycle, see Fig. 3k and Supplementary Fig. 14a). Hence, FeP–CoP/NC catalyst on each electrode was subjected to both anodic and cathodic bias. Notably, the current density of the cell stabilized at ca. ±43 mA cm^-2^ in both the “positive” and “negative” cycle. To avoid the influence of the evolved gas around the electrode on the next cycle, we also assembled an H-type cell comprising FeP–CoP/NC on carbon cloth as both electrodes. Similar cyclic chronoamperometric studies were performed (±1.68 V, 3.3 h per cycle, see Fig. 3l and Supplementary Fig. 14b). Although the current density remained stable during all cycles, the value dropped to ±10 mA cm^-2^ due to the improved ohmic resistance of the cell. We concluded that FeP–CoP/NC on carbon cloth were excellent bifunctional electrode, yet the high ohmic loss of the conventional alkaline water electrolysis cell restricted its performance in practice.

To understand the origin of the bifunctional activity, we performed X-ray photoelectron spectroscopy (XPS). The deconvoluted Fe 2*p* core-level spectra of FeP–CoP/NC before and after HER/OER cycle is shown in Supplementary Fig. 15a. The peaks at 707.2 and 720.1 eV of the fresh sample correspond to the formation of phosphide^49,50^. They disappeared completely after the reaction, plausibly due to the oxidation and the subsequent formation of iron oxide and hydroxide. CoP also experienced oxidation and the formation of oxyhydroxide during the OER cycle, in agreement with scenario observed in the literature, yet the phosphide peaks at 779.4 and 794.5 eV were still observed in Supplementary Fig. 15b^51–56^. This might be ascribed to the electronegativity difference of CoP and FeP. When connected with each other in the electrolyte, the more electropositive CoP was largely protected and stabilized whereas FeP was oxidized. Because both CoP and FeP were uniformly distributed in the nanoparticle (cf. the TEM microscopic analysis), the remaining CoP quantum dots were still capable of effectively catalyzing HER after OER cycle. Therefore, we inferred the origin of the bifunctionality: the in-situ generated mixed Fe and Co (oxy)hydroxides contributed to OER whereas the residual CoP quantum dots were responsible for HER during the continuous cyclic operation.

### Performance of MFE

**Fig. 4:**
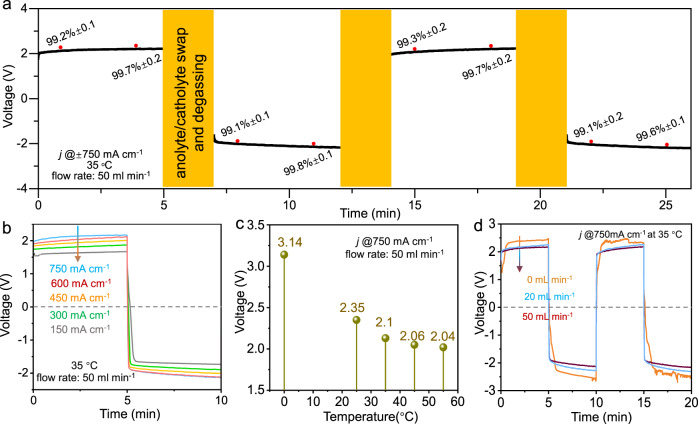
The performance of MFE in overall water splitting. **a** A typical operation sequences during the cyclic test, the H_2_ purity was determined at the time with the red dots; the influence of **b** current densities, **c** temperature, and **d** flow rate on the potential bias, other than specified, current density is 750 mA cm^−2^, temperature is 35 °C and flow rate is 50 ml min^−1^, anolyte/catholyte swap is not applied for simplicity. Both anolyte and catholyte are 1.0 M KOH solutions.

The FeP–CoP/NC bifunctional catalyst was screen printed on the carbon cloth (CC) as the working electrode of the MFE. Before testing, both compartments of the MFE was filled up with 1.0 M KOH solution. Selecting this relatively low concentration (compared with 30–40% KOH solution in industrial alkaline water electrolyzer) was to ensure the stability of phosphide electrocatalyst in the OER cycle against oxidation. The auxiliary electrodes were activated in-situ to form the Ni(OH)_2_/NiOOH redox couple (see Fig. 1, more details can be found in the SI). Figure 4b shows the cyclic galvanostatic curves of MFE at ±750 mA cm^-2^. Each cycle lasted for 10 min to avoid the over discharging of the auxiliary electrode (the influence of cycle time can be found in Supplementary Fig. 16). The recorded potential bias, stabilized at 2.1 V, was highly symmetric for all the “positive” and “negative” cycles. When the current polarity was swapped, the flowing anolyte and catholyte were also exchanged during the 2 min interval (for simplicity, this interval was omitted in the plots below). The H_2_ purity, determined at all the sampling time (see red dots in Fig. 4a), maintained >99.1% in all the investigated cycles.

MFE can work at different current densities (see Fig. 4b). The applied voltages were 1.62, 1.78, 1.92, 2.01, and 2.11 V to reach the current densities of 150, 300, 450, 600, and 750 mA cm^-2^, respectively. The low overpotentials indicated the excellent bifunctional activity of the catalyst, comparable with the PEMEC while outperforming the classic alkaline water and conventional decoupled electrolyzer, using redox couples (CE, see Supplementary Figs. 18–19). We also investigated the influence of the compartment temperature on water splitting, satisfactory applied voltages were recorded above 35 °C (Fig. 4c). This low operating temperature promised to significantly save the heating energy. The flow rate of anolyte/catholyte was another vital factor controlling the reaction kinetics (see Fig. 4d). Thanks to the facilitated mass transport at 50 ml min^-1^, the polarization loss was greatly reduced and stabilized.

**Fig. 5:**
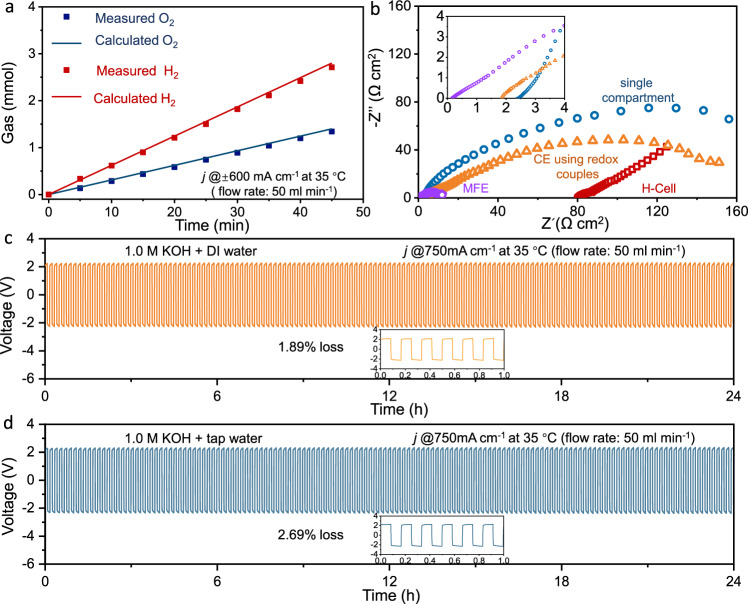
Stability test of MFE in overall water splitting. **a** Measured H_2_ and O_2_ yields using Archimedes’ method, solids lines represent the gas evolution at 100% Faradaic efficiency; **b** EIS spectra of cells with different architectures, all electrodes are employed with FeP–CoP/NC catalysts; cyclic stability test of MFE in **c** deionized water and **d** tap water electrolyte, the precipitation in the tap water electrolyte, after adding KOH, has been removed; anolyte/catholyte swap is not applied for simplicity.

We also used Archimedes’ method to quantify the generated gas volume over time at 200 mA (see Fig. 5a). After 45 min, 2.77 mmol of H_2_ has been measured, which was close to the theoretical value (2.8 mmol), the calculated Faradaic efficiency was 96.5%. The loss was ascribed to the charge/discharge of the auxiliary electrode and the polarization of the electrode. Figure 5b compares the electrochemical impedance spectra (EIS) of electrolyzers with various architectures at open circuit voltage (OCV). The area specific resistance of MFE was as low as 0.17 Ω cm^2^, comparable with that of PEMEC yet much lower than that of single-compartment cell, H-cell and the control cell (NiOOH/Ni(OH)_2_ redox couple was used yet no flow or sandwiched electrode structure, see details in Supplementary Figs. 18 and 22).

Importantly, the overall water splitting in the MFE was performed equally well in both deionized water and regular tap water containing various metal cations (see Fig. 5c, d and Supplementary Fig. 24). After 24 h test at 750 mA cm^-2^ in deionized water electrolyte, little degradation has been observed. The hardness of the used tap water was 179 mg L^-1^ (equivalent concentration as calcium carbonate, mainly originated from Ca^2+^ and Mg^2+^). Other soluble and solid contaminants can be found in Supplementary Table 4. Albeit that these cations are known to be detrimental to PEMEC water electrolysis, it seems that MFE is not affected. The potential bias at 750 mA cm^-2^ was increased slightly by 60–70 mV compared with that in the deionized water, yet was also essentially stable during 24 h test. In Supplementary Fig. 25, we also showed a 500 h stable operation of MFE in tap water electrolyte. The overpotential at 750 mA cm^-2^ increased by 8.95% after 500 h longevity test, suggesting that both the working and auxiliary electrodes were robust under the operating conditions.

## Discussion

Our membrane-free flow electrolyzer can generate H_2_ with high purity (>99.1%). The sandwich-like architecture enabled low ohmic loss, whereas the cost-effective FeP–CoP/NC bifunctional catalysts guaranteed the efficient and stable overall water splitting in MFE in cyclic mode. The highest current density reached 750 mA cm^-2^ under the potential bias of 2.1 V and ambient conditions, in both deionized and tap water electrolyte. Scaling up of this cell technology requires a complete techno-economic analysis, particularly taking into account the auxiliary electrode cost. In addition, the redox stability of the working and auxiliary electrodes in the tap water electrolyte should be further evaluated. Nevertheless, this work demonstrates the opportunity of combining the advantages of conventional alkaline electrolyzer with low materials/operation costs and PEMEC with high current densities, opening a path for sustainable hydrogen generation.

## Methods

### Materials and reagents

All chemical reagents and materials, including Co(NO_3_)_2_·6H_2_O, Fe(NO_3_)_3_·9H_2_O, NaH_2_PO_2_·H_2_O, (MgCO_3_)_4_Mg(OH)_2_, N(CH_2_COOH)_3_ were used without purification. Nafion solution was obtained from E.I. Du Pont. Ni(OH)_2_, CoO, polytetrafluoroethylene (PTFE), porous separator and carboxymethylcellulose (CMC) were provided by NewCom energy material store. Deionized water (DI) was used for the materials synthesis.

### Preparation of FeP-CoP/NC and the control samples (FeP/NC, CoP/NC, FeP-CoP, and FeP-CoP/C)

Preparation of porous N doped carbon (NC). The NC was synthesized according to our previous work^57^.

#### Preparation of FeP-CoP/NC

We used the classic infiltration method. 60 mg NC was added to the solution containing 2 mmol Fe(NO_3_)_3_·9H_2_O and 2 mmol Co(NO_3_)_2_ ·6H_2_O dissolved in 15 mL DI water. The resulting suspension was stirred at 65 °C for 12 h until water evaporated completely. The obtained powder was reduced in a tubular furnace at 550 °C for 2 h in 5% H_2_ atmosphere to form Fe–Co bimetallic alloy/NC. Then, Fe–Co bimetallic alloy/NC and NaH_2_PO_2_·H_2_O were placed at two separate positions in a tube with the stream of Ar. NaH_2_PO_2_·H_2_O was placed at the upstream side. The mass ratio of NaH_2_PO_2_ · H_2_O and Fe–Co bimetallic alloy/NC was 5:1. The sample was heated up to 300 °C for 120 min to form FeP–CoP/NC. FeP/NC, CoP/NC, FeP–CoP, and FeP-CoP/C were prepared using the same approach.

### Preparation of the modified electrode

The glassy carbon electrode (GC) (Φ = 3 mm, *A*: 0.0706 cm^2^) was polished with 0.3 and 0.05 μm alumina powders, and rinsed with acetone, ethanol, and DI water. GC was calibrated in 1.0 mM K_3_Fe(CN)_6_ + 1.0 mM K_4_Fe(CN)_6_ + 0.1 M KCl (see CV in Supplementary Fig. 1). The catalyst ink contained 3 mg catalyst, 50 μL 5 wt% Nafion solution, 50 μL DI water and 900 μL ethanol. After sonication for 2 h, 8 μL of catalyst ink was drop-casted on the polished GC (mass loading 0.340 mg cm^-2^) and dried in air.

### Preparation of FeP–CoP/NC coated carbon cloth electrodes (FeP–CoP/NC/CC)

FeP–CoP/NC was brush-painted on the cleaned carbon cloth (2 × 2 cm), the mass loading of FeP–CoP/NC was 3.17 mg cm^−^^2^. The obtained FeP–CoP/NC/CC was dried in a vacuum oven overnight to remove all the organic solvents.

### Preparation of Ni(OH)_2_ auxiliary electrode (AE)

1.32 g Ni(OH)_2_, 0.2493 g CoO, 0.0665 g polytetrafluoroethylene (PTFE), and 0.016625 g carboxymethylcellulose (CMC) were mixed with water to form the slurry. The slurry was sequentially brush painted on a piece of cleaned Ni foam (2 × 2 cm), dried and pressed under a pressure of 20 MPa. The mass loading of Ni(OH)_2_ was 330 mg cm^-2^. To prevent the delamination of the coated film under redox cycle in reaction, this coated Ni piece was then sandwiched by two pristine Ni foam piece, as shown in Supplementary Fig. 2, and pressed again under a pressure of 20 MPa to form the final AE.

### Assembly of MFE

Both compartments of MFE were comprised of the endplates, current collectors, a bifunctionally active electrode (1 × 0.333 cm), a porous separator (130 μm-thick) and an AE (1 × 2 cm), respectively. Two AEs were connected by a copper strip. Two peristaltic pumps were used to enable the flow of electrolyte.

### Materials characterizations

X-ray diffraction (XRD) patterns were obtained by using a D8 Advance X-ray diffractometer with Cu Kα radiation (*λ* = 1.5418 Ǻ). Diffraction patterns were collected by step scanning in the 2*θ* range of 20–80° with an interval of 0.02°. The morphologies were investigated by a field-emission scanning electron microscopy (FE-SEM, Hitachi S-4800). Transmission electron microscopy (TEM) and elemental mapping were collected by using a TEM (JEOL, JEM-2100F) equipped with an energy-disperse X-ray spectrometer (EDX) attachment. The surface analysis was performed by X-ray photoelectron spectroscopy (XPS, ESCALab 250Xi Thermo Scientific) with Al Kα (hν = 1486.6 eV) radiation. All the binding energies were calibrated to the adventitious C 1 *s* peak at 284.8 eV. Brunauer–Emmett–Teller (BET) were obtained by a Belsorp-Mini II (BEL Japan Inc., Japan) at 77 K in N_2_. Gas purity was measured using an online gas chromatography (Fuli GC9730).

### Electrochemical characterizations

All electrochemical measurements were performed using a potentiostat (Autolab pgstat M204). In a typical three-electrode system, 1 M KOH solution was used as the electrolyte, a platinum plate (0.5 × 0.5 cm) was used as the counter electrode, a saturated calomel electrode (SCE) was used as the reference electrode. In the OER and HER measurement, the electrolyte was saturated by O_2_ and H_2_ in advance, respectively. Linear sweep voltammetry (LSV) and cyclic voltammetry (CV) were performed at 5 mV s^-1^. The Tafel slope was calculated using the Tafel equation:

1 $${\rm{\eta }}={\rm{blog}}({\rm{j}})+{\rm{a}}$$

(*η*, *b*, and *j* represent the overpotential, Tafel slope, and current density, respectively), and a scan rate of 5 mV s^−1^ was used to obtain the polarization curves for Tafel plots. Electrochemical impedance spectra (EIS) were recorded at frequencies ranging from 10^5^ Hz to 0.1 Hz. The long-term stability tests of OER and HER were performed by chronoamperometry and chronopotentiometry. All the potentials reported in our work were relative to the reversible hydrogen electrode (RHE) using the conversion equation below:

2 $${{\rm{E}}}_{{\rm{RHE}}}={{\rm{E}}}_{{\rm{SCE}}}+0.059\times {\rm{pH}}+0.242{\rm{V}}.$$

### Faradaic efficiency measurement

We used Archimedes’ method to measure the volume of the collected gas at several time points during electrolysis. Faradaic efficiency (FE) was calculated according to the following equation:

3 $${FE}=\frac{{n({{\mathrm{gas}}})}_{{{\mathrm{measured}}}}}{{n({{\mathrm{gas}}})}_{{{\mathrm{calculated}}}}}=\frac{P{{\rm{\cdot }}V({{\mathrm{gas}}})}_{{{\mathrm{measured}}}}/{\rm{R}}T}{Q/z{\rm{F}}}$$

wherein *P* is the pressure (1 atm), *V* is the volume of the generated gas, R is gas constant (8.314 J mol^-1^ K^-1^), *T* is temperature (298 K), *Q* = ∫*I*dt is the charge, *z* is the stoichiometric charge number (2 electrons per H_2_ molecule and 4 electrons per O_2_ molecule), and F is Faraday constant (F = 96485.34 C mol^-1^).

### Activation of AEs

The NiOOH/Ni(OH)_2_ redox couple was generated in situ. In the original MFE assembly as shown in Supplementary Fig. 5, both AEs contained Ni(OH)_2_ only. The activation process was carried out by periodically applying ±250 mA current to MFE (30 min per cycle for 40 cycles) to generate NiOOH on AE:

4 $${Ni}{\left({OH}\right)}_{2}+O{H}^{-}\to {NiOOH}+{H}_{2}O+{e}^{-}$$

## Supplementary information

Supplementary Information

## Data Availability

The data that supports the plots of this paper and other findings of this study are available from the corresponding authors upon reasonable request.

## References

[1] Huang S (2017). N-, O-, and S-tridoped carbon-encapsulated Co_9_S_8_ nanomaterials: efficient bifunctional electrocatalysts for overall water splitting. Adv. Funct. Mater..

[2] Niether C (2018). Improved water electrolysis using magnetic heating of FeC–Ni core–shell nanoparticles. Nat. Energy.

[3] Zhang Q, Bedford NM, Pan J, Lu X, Amal R (2019). A fully reversible water electrolyzer cell made up from FeCoNi (Oxy)hydroxide atomic Layers. Adv. Energy Mater..

[4] Guo M (2020). In situ conversion of metal (Ni, Co or Fe) foams into metal sulfide (Ni_3_S_2_, Co_9_S_8_ or FeS) foams with surface grown N-doped carbon nanotube arrays as efficient superaerophobic electrocatalysts for overall water splitting. J. Mater. Chem. A.

[5] Sun H (2020). Boosting activity on Co_4_N porous nanosheet by coupling CeO_2_ for efficient electrochemical overall water splitting at high current densities. Adv. Funct. Mater..

[6] Suryanto BHR, Wang Y, Hocking RK, Adamson W, Zhao C (2019). Overall electrochemical splitting of water at the heterogeneous interface of nickel and iron oxide. Nat. Commun..

[7] Hui L (2018). Overall water splitting by graphdiyne-exfoliated and -sandwiched layered double-hydroxide nanosheet arrays. Nat. Commun..

[8] van Troostwijk A, Deiman JR (1789). Sur une manière de décomposer l’Eau en Air inflammable et en Air vital. Obs Phys..

[9] Trasatti S (1999). Water electrolysis: who first?. J. Electroanal. Chem..

[10] Ursua A, Gandia LM, Sanchis P (2012). Hydrogen production from water electrolysis: current status and future trends. Proc. IEEE.

[11] You B, Sun Y (2016). Chalcogenide and phosphide solid‐state electrocatalysts for hydrogen generation. Chempluschem.

[12] Lin Y, Gao Y, Fan Z (2017). Printable fabrication of nanocoral‐structured electrodes for high‐performance flexible and planar supercapacitor with artistic design. Adv. Mater..

[13] Li D, Park EJ, Zhu W, Shi Q, Kim YS (2020). Highly quaternized polystyrene ionomers for high performance anion exchange membrane water electrolysers. Nat. Energy.

[14] Liu C (2018). Performance enhancement of PEM electrolyzers through iridium-coated titanium porous transport layers. Electrochem. Commun..

[15] Vengatesan S, Santhi S, Jeevanantham S, Sozhan G (2015). Quaternized poly (styrene-co-vinylbenzyl chloride) anion exchange membranes for alkaline water electrolysers. J. Power Sources.

[16] Smith DW (2013). A microblock ionomer in proton exchange membrane electrolysis for the production of high purity hydrogen. Macromolecules.

[17] Chen L, Dong X, Wang Y, Xia Y (2016). Separating hydrogen and oxygen evolution in alkaline water electrolysis using nickel hydroxide. Nat. Commun..

[18] Araya SS, Kær SK (2019). Long-term contamination effect of iron ions on cell performance degradation of proton exchange membrane water electrolyser. J. Power Sources.

[19] Tang H, Peikang S, Jiang SP, Wang F, Pan M (2007). A degradation study of nafion proton exchange membrane of PEM fuel cells. J. Power Sources.

[20] Leng Y (2012). Solid-state water electrolysis with an alkaline membrane. J. Am. Chem. Soc..

[21] Zhou H (2018). Water splitting by electrolysis at high current densities under 1.6 volts. Energy Environ. Sci..

[22] Firouzjaie HA, Mustain WE (2019). Catalytic advantages, challenges and priorities in alkaline membrane fuel cells. ACS Catal..

[23] Landman A (2017). Photoelectrochemical water splitting in separate oxygen and hydrogen cells. Nat. Mater..

[24] Gillespie MI, Kriek RJ (2018). Scalable hydrogen production from a mono-circular filter press divergent electrode-flow-through alkaline electrolysis stack. J. Power Sources.

[25] Talabi OO, Dorfi AE, O’Neil GD, Esposito DV (2017). Membraneless electrolyzers for the simultaneous production of acid and base. Chem. Commun..

[26] Yang F, Kim MJ, Brown M, Wiley BJ (2020). Alkaline water electrolysis at 25 A cm^-2^ with a microfibrous flow‐through electrode. Adv. Energy Mater..

[27] Clarke RE, Giddey S, Badwal S (2010). Stand-alone PEM water electrolysis system for fail safe operation with a renewable energy source. Int. J. Hydrog. Energy.

[28] Amstutz V (2014). Renewable hydrogen generation from a dual-circuit redox flow battery. Energy Environ. Sci..

[29] Goodwin S, Walsh DA (2017). Closed bipolar electrodes for spatial separation of H_2_ and O_2_ evolution during water electrolysis and the development of high-voltage fuel cells. ACS Appl. Mat. Interfaces.

[30] Dotan H (2019). Decoupled hydrogen and oxygen evolution by a two-step electrochemical–chemical cycle for efficient overall water splitting. Nat. Energy.

[31] Li Y (2020). Implanting Ni-O-VOx sites into Cu-doped Ni for low-overpotential alkaline hydrogen evolution. Nat. Commun..

[32] Lagadec MF, Grimaud A (2020). Water electrolysers with closed and open electrochemical systems. Nat. Mater..

[33] Ben-Jacob E, Garik P (1990). The formation of patterns in non-equilibrium growth. Nature.

[34] Huang J (2021). Decoupled amphoteric water electrolysis and its integration with Mn–Zn battery for flexible utilization of renewables. Energy Environ. Sci..

[35] Gillespie MI, Kriek RJ (2017). Hydrogen production from a rectangular horizontal filter press divergent electrode-flow-through (DEFT™) alkaline electrolysis stack. J. Power Sources.

[36] You B, Sun Y (2018). Innovative strategies for electrocatalytic water splitting. Acc. Chem. Res..

[37] Xiang C, Papadantonakis KM, Lewis NS (2016). Principles and implementations of electrolysis systems for water splitting. Mater. Horiz..

[38] Gillespie MI, van der Merwe F, Kriek RJ (2015). Performance evaluation of a membraneless divergent electrode-flow-through (DEFT) alkaline electrolyser based on optimisation of electrolytic flow and electrode gap. J. Power Sources.

[39] Li Z (2017). Core-shell structured CoP/FeP porous microcubes interconnected by reduced graphene oxide as high performance anodes for sodium ion batteries. Nano Energy.

[40] Zhou B, Yan F, Li X, Zhou J, Zhang W (2019). An interpenetrating porous organic polymer as a precursor for FeP/Fe_2_P‐embedded porous carbon toward a pH‐universal ORR catalyst. Chemsuschem.

[41] Liu T (2019). CoP‐doped MOF‐based electrocatalyst for pH‐universal hydrogen evolution reaction. Angew. Chem. Int Ed..

[42] Tang C (2017). Fe-doped CoP nanoarray: a monolithic multifunctional catalyst for highly efficient hydrogen generation. Adv. Mater..

[43] Li H, Xu SM, Yan H, Yang L, Xu S (2018). Cobalt phosphide composite encapsulated within N, P‐doped carbon nanotubes for synergistic oxygen evolution. Small.

[44] Lv Y (2020). Fe‐Co alloyed nanoparticles catalysed efficient hydrogenation of cinnamaldehyde to cinnamyl alcohol in water. Angew. Chem. Int. Ed..

[45] Liu T, Wang K, Du G, Asiri AM, Sun X (2016). Self-supported CoP nanosheet arrays: a non-precious metal catalyst for efficient hydrogen generation from alkaline NaBH4 solution. J. Mater. Chem. A.

[46] Yao C (2020). Atomically-precise dopant-controlled single cluster catalysis for electrochemical nitrogen reduction. Nat. Commun..

[47] Wang J (2021). Heteroatom‐doping of non-noble metal‐based catalysts for electrocatalytic hydrogen evolution: an electronic structure tuning strategy. Small Methods.

[48] Hua B (2017). Stabilizing double perovskite for effective bifunctional oxygen electrocatalysis in alkaline conditions. Chem. Mater..

[49] Xu J (2018). Boosting the hydrogen evolution performance of ruthenium clusters through synergistic coupling with cobalt phosphide. Energy Environ. Sci..

[50] Zhang Q (2018). Iron-doped NiCoP porous nanosheet arrays as a highly efficient electrocatalyst for oxygen evolution reaction. ACS Appl. Energy Mater..

[51] Jiao L, Zhou YX, Jiang H (2016). Metal-organic framework-based CoP/reduced graphene oxide: high-performance bifunctional electrocatalyst for overall water splitting. Chem. Sci..

[52] Yan X (2020). “Nano-garden cultivation” for electrocatalysis: controlled synthesis of nature-inspired hierarchical nanostructures. J. Mater. Chem. A.

[53] Boppella R, Tan J, Yang W, Moon J (2019). Homologous CoP/NiCoP heterostructure on N-doped carbon for highly efficient and pH-universal hydrogen evolution electrocatalysis. Adv. Funct. Mater..

[54] Guan C (2018). Hollow Mo-doped CoP nanoarrays for efficient overall water splitting. Nano Energy.

[55] Pan Y (2018). Core-shell ZIF-8@ZIF-67 derived CoP nanoparticles-embedded N-doped carbon nanotube hollow polyhedron for efficient over-all water splitting. J. Am. Chem. Soc..

[56] Liu T (2017). Nickel-Cobalt phosphide nanowires supported on Ni foam as a highly efficient catalyst for electrochemical hydrogen evolution reaction. Int. J. Hydrog. Energy.

[57] Eisenberg D (2016). A simple synthesis of an N-doped carbon ORR catalyst: hierarchical micro/meso/macro porosity and graphitic shells. Chem. Eur. J..

