# Supplementary material for: A membrane-free flow electrolyzer operating at high current density using earth-abundant catalysts for water splitting
Source: Nat Commun. 2021 Jul 6;12:4143. doi: 10.1038/s41467-021-24284-5 (PMC8260598; doi:10.1038/s41467-021-24284-5)
Supplement: Supplementary file 1 — Supplementary Information [file 41467_2021_24284_MOESM1_ESM.pdf]

Supplementary information

A membrane-free flow electrolyzer operating at high current density using earth-abundant catalysts for water splitting

Xiaoyu Yan,[1,3] Jasper Biemolt,[2] Kai Zhao,[1,3] Yang Zhao,[1] Xiaojuan Cao,[1,3] Ying Yang,[4] Xiaoyu Wu,[1,3] Gadi Rothenberg,[2] and Ning Yan [1,2,3,*]

[1] School of Physics and Technology, Wuhan University, Wuhan, China.

[2] Van`t Hoff Institute for Molecular Sciences (HIMS), University of Amsterdam, Amsterdam, The Netherlands.

[3] Suzhou Institute of Wuhan University, Suzhou, China.

[4] Hubei Key Laboratory of Theory and Application of Advanced Materials Mechanics, Wuhan University of Technology, Wuhan, China.

*To whom the correspondence should be addressed: ning.yan@whu.edu.cn

Supplementary information includes:

1. Supplementary Tables 3

2. Supplementary Figures 7

1. Supplementary Tables

Supplementary Table 1: Comparison of OER overpotentials with selected high-performance electrocatalysts at 10 mA cm^{-2} and 50 mA cm^{-2} in 1.0 M KOH solutions.

Catalyst	$\eta_{=10}$ (mV)	$\eta_{=50}$ (mV)	References
NiMoOx/NiMoS	186	206	Nat. Commun., 2020, 11, 5462-5474
**FeP-CoP/NC/CC**	**201**	**319**	**This work**
p-$Cu_{1-x}NNi_{3-y}$/FeNiCu	280	352	Nat. Commun., 2018, 9, 2326
Ni_3FeN	280	345	Adv. Energy Mater., 2016, 6, 1502333
Ni_3N nanosheets	290	333	Angew. Chem. Int. Ed., 2016, 55, 8670-8674
Ni_2P	290	n.g.	Energy Environ. Sci., 2015, 8, 2347-2351.
Co-Bi NS/G nanosheets	290	345	Angew. Chem. Int. Ed., 2016, 55, 2488
CoM-P-3DHFLMs	292	400	Appl. Catal. B, 2019, 249 147–154
Amorphous CoSe film	292	351	Chem. Commun., 2015, 51, 16683-16686
Zn doped CoO	293	341	ChemCatChem, 2019, 11, 1480–1486
α-$Co_4Fe(OH)x$	295	350	J. Mater. Chem., A 2017, 5, 1078
$SCFW_{0.4}$	296	n.g.	J. Mater. Chem. A, 2018, 6, 9854-9859
$CoSe_{0.85}$	300	325	Adv. Mater., 2016, 28, 77-85
CoFe LDH-F	300	n.g.	Adv. Mater. Interfaces, 2019, 10, 1409
boronized Ni	300	330	J. Mater. Chem. A, 2019, 7, 5288-5294
CoFe LDH/NF	300	390	ChemPlusChem, 2017, 82, 483
Poled Co2	320	n.g.	Nat. Commun., 2016, 8, 14430
CoMnP	330	n.g.	J. Am. Chem. Soc., 2016, 138, 4006-4009
Co_4N	330	370	J. Am. Chem. Soc., 2015, 137, 4119-4125
p-SnNiFe	350	445	Nat. Commun., 2017, 8, 934
NiFe@NC	360	n.g.	Nano Energy., 2017, 39, 245–252

Note: n.g. indicates not given or obtainable in the literature.

Supplementary Table S2: Comparison of HER overpotentials of FeP-CoP/NC/CC with selected high-performance electrocatalysts at 10 mA cm^{-2} and 50 mA cm^{-2} in 1.0 M KOH solutions.

Catalyst	η=10 (mV)	η=50 (mV)	References
**FeP-CoP/NC/CC**	**52**	**81**	**This work**
Ir-NSG	18.5	n.g	Nat. Commun., 2020, 11, 1-11
NiMoOx/NiMoS	38	67	Nat. Commun., 2020, 11, 5462-5474
Pt-Ni NWs-S/C	59	n.g	Nat. Commun., 2017, 8, 14580
Co-Fe-P	66	210	Nano Energy., 2019，56，225-233
FLNPC@MoP-NC/MoP-C/CF	69	156	Adv. Funct. Mater., 2018, 28
Mn-Co-P/Ti	76	121	ACS Catal., 2017, 7, 98–102
np-$(Co_{0.52}Fe_{0.48})_2P$	79	98	Energy Environ. Sci., 2016, 9, 2257
CoP/NPC/TF	80	148	J. Mater. Chem. A, 2018, 6, 6282
V-Ni_2P NSAs/CC	85	275	Nanoscale, 2019, 11, 4198-4203
$Ni_{0.33}Co_{0.67}S_2$	88	181	Adv. Energy Mater., 2015, 5, 1402031
CoP/Ti	90	127	Chem. Mater., 2014, 26, 4326
NFP/C-3	95	150	Sci. Adv., 2019, 5, 6009
$FePSe_3$/NC	118	175	Nano Energy., 2019, 57, 222–229
$CoMoS_3$ nanotubes	133	270	J. Mater. Chem. A, 2017, 5, 11309
CoS_2 NW	145	190	J. Am. Chem. Soc., 2014, 136, 10053
CoP/Co_2P/Co	160	n.g.	ACS Appl. Mater., Interfaces 2018, 10, 15673
NiS_2–MoS_2 hetero-nanowires	160	210	J. Mater. Chem. A, 2016, 4, 13439–13443
Co/CoP-5	175	320	Adv. Energy Mater., 2017, 7, 1602355

Note: n.g. indicates not given or obtainable in the literature.

Supplementary Table S3: Comparison of the applied voltage FeP-CoP/NC/CC with selected high-performance electrocatalysts at 10 mA cm^{-2} and 50 mA cm^{-2} in 1.0 M KOH.

Catalyst	$V_{=10}$ (V)	$V_{=50}$ (V)	References
NiMoOx/NiMoS	1.46	1.55	Nat. Commun., 2020, 11, 5462-5474
Ni-ZIF/NiB	1.54	1.73	Adv. Energy Mater., 2020 10, 1902714
**FeP-CoP-CN/CC**	**1.56**	**1.67**	**This work (no iR compensation)**
Mo-Co_9S_8@C	1.56	1.95	Adv. Energy Mater., 2020 10, 1903137
Ni/$Ni(OH)_2$	1.59	1.68	Adv. Mater., 2020, 32, 1906915
O-CoP	1.61	1.75	Adv. Funct. Mater., 2020, 30, 1905252
CoP flower/CC	1.61	1.71	J. Mater. Chem. A, 2020, 8, 7626-7632
Fe(0.2)/Ni-M@C-400-2h	1.62	1.75	ACS Appl. Mater. Interfaces, 2020, 12, 55782–55794
IrP_2/NPC	1.62	1.78	ACS Appl. Mater. Interfaces 2019, 11, 16461-16473
CoP/Ni foam	1.62	1.69	Adv. Funct. Mater., 2015, 25,7337–7347
FCP@NG	1.63	1.83	Nanoscale, 2019, 11, 12837–12845
CoFeZr oxides/NF	1.63	1.75	Adv. Mater. 2019, 31, 1901439
Ni-Pi/CF	1.63	2.15	Adv. Funct. Mater., 2016, 26, 4067–4077
Ni_2Fe_1-O	1.64	1.79	Adv. Energy Mater., 2018, 81701347
MoS_2-NiS_2/NGF	1.64	n.g.	Appl. Catal., B 2019,254, 15–25
$NiCo_2S_4$-4	1.64	1.78	Adv. Funct. Mater. 2019, 29, 1807031
CoP/NCNHP	1.64	1.77	Adv. Mater. 2019, 31, 1808167
CVN/CC	1.64	1.92	Appl. Catal., B 2019, 241, 521–527
Co-P film	1.64	1.71	Angew. Chem. Int. Edl. 2015,54, 6251-6254
Ni foam–supported Ni/Mo_2C (1:2)-NCNFs	1.64	n.g.	Adv. Energy Mater. 2019, 9, 1803185
Ni_3S_2	1.65	1.78	Nanoscale, 2019, 11, 5646-5654
$Co_{0.85}Se$/NiFe-LDH	1.67	n.g.	Energ. Environ. Sci., 2016, 9, 478-483
Co_1Mn_1CH/NF	1.68	1.86	J. Am. Chem. Soc., 2017, 139,8320–8328
Co_9S_8/WS_2	1.68	1.82	J. Mater. Chem. A, 2017, 5, 23361-23368
$Fe_{0.4}Co_{0.6}$	1.68	1.72	Nano Energy, 2017, 38, 576-584
CoP/rGO-400	1.70	n.g.	Chem. Sci., 2016, 7, 1690-1695
NESSP//NESS	1.74	1.92	Adv. Mater., 2017, 29, 1702095
CC/CNTs@$CoS_{0.74}Se_{0.52}$	1.74	1.88	ChemSusChem, 2019, 12, 3792 – 3800
NiFeOF/Ni foam	1.80	n.g.	ACS Catal., 2017, 7, 8406–8412
PPy/FeTCPP/Co	1.81	n.g.	Adv. Funct. Mater., 2017, 27, 1606497
NiCoFeB	1.81	1.89	Small, 2019, 15, 1804212

Note: n.g. indicates not given or obtainable in the literature.

Supplementary Table S4: Minerals in the tap water used in this work

Sequence number	Test index (mg L^-)	Index limit (mg L^-)	Test result (mg L^-)
1	total hardness (Ca^{2+}+Mg^{2+})	450	138~179
2	solid solution	1000	161~240
3	$M(SO_4)_X$	250	36.2~44.7
4	RClx	250	21.5~30.4
5	NO_3-N	10	1.59~2.11
6	$R(MnO_4)x$	5	1.26~2.56
7	Fe^{x+}	0.3	0.013~0.017
8	anion detergent	0.3	0.05
9	Al^{3+}	0.2	0.044~0.107
10	Mn^{3+}	0.1	0.0001~0.0072
11	RFx	1.0	0.16~0.22
12	Cu^{x+}	1.0	0.0005~0.0024
13	Zn^{2+}	1.0	0.001~0.009
14	CH_3Cl	0.06	0.023
15	R(CN)x	0.05	0.002
16	$Cr_2O_7^-$	0.05	0.004
17	As^{x+}	0.01	0.0010~0.0018
18	Se^{x+}	0.01	0.0005~0.0006
19	Pb^{x+}	0.01	0.0001
20	Cd^{x+}	0.005	0.0001
21	CCl_4	0.002	0.0001
22	Hg^{x+}	0.001	0.00005

2. Supplementary Figures

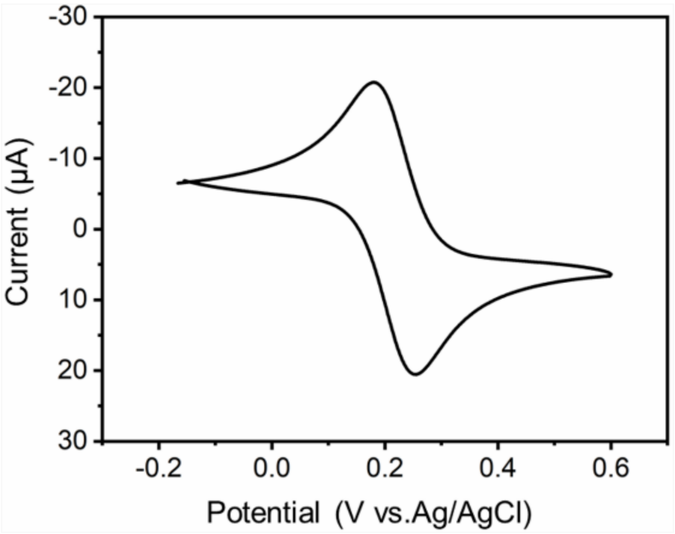

Supplementary Figure 1: CV of GC electrode in 1.0 mM $K_3Fe(CN)_6$ + 1.0 mM $K_4Fe(CN)_6$ + 0.1 M KCl at 5 mV s^{-1} for calibration.

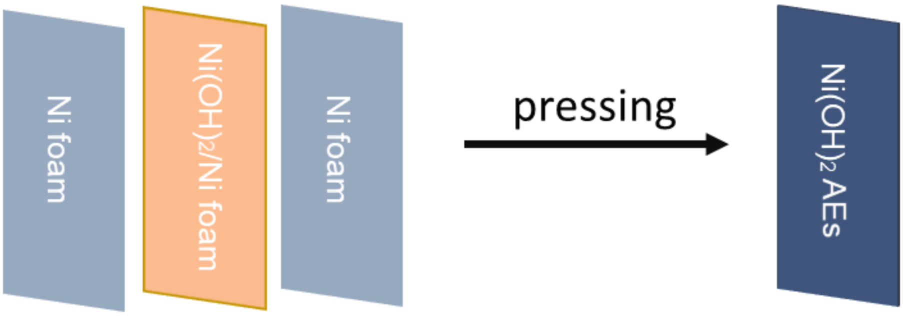

Supplementary Figure 2: The preparation process and structure of the AE.

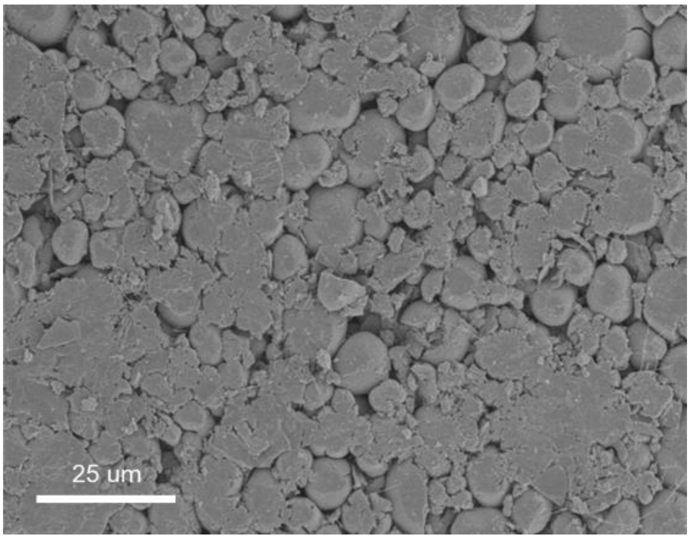

Supplementary Figure 3: SEM image of $Ni(OH)_2$ in a AE.

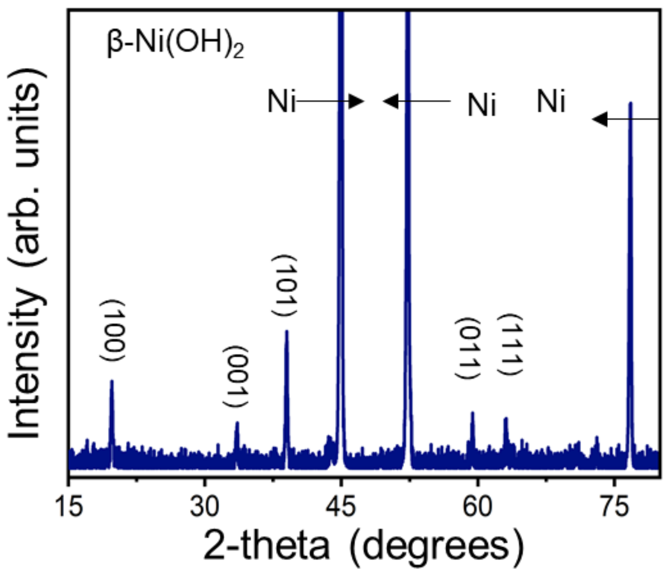

Supplementary Figure 4: XRD pattern of AE; three main peaks corresponds to the Ni form matrix where as the remaining peaks belong to $Ni(OH)_2$.

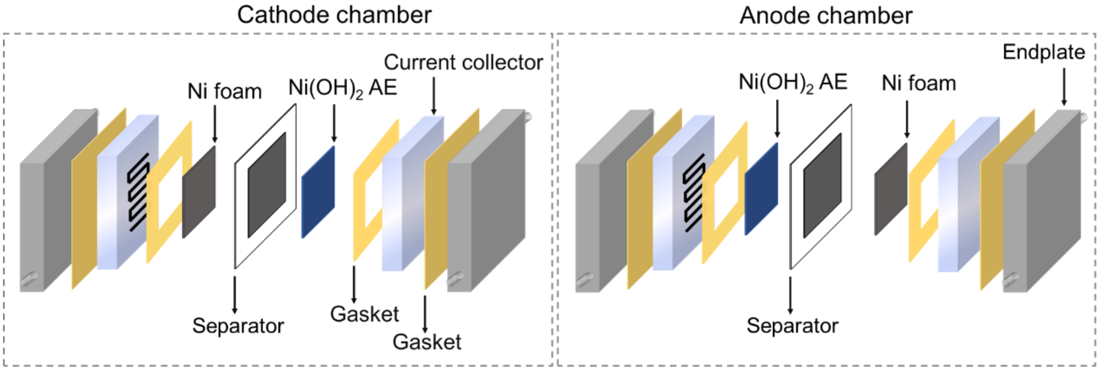

Supplementary Figure 5: The original assembly of MFE before activation.

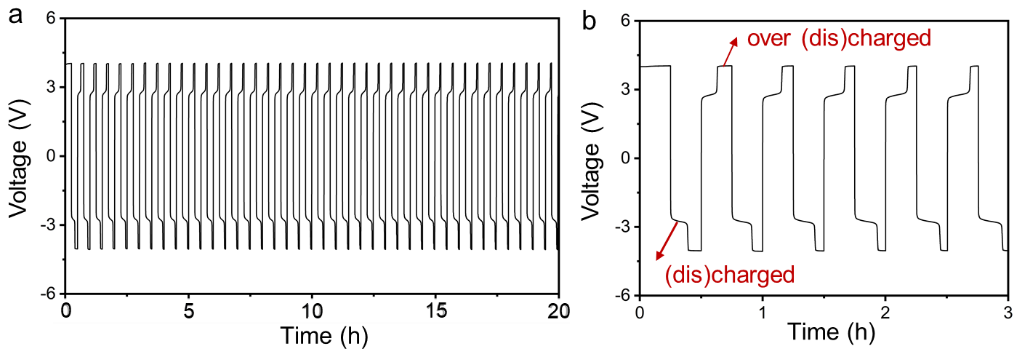

Supplementary Figure 6: Activation process of AEs in MFE, current is ±250 mA, temperature is 35 °C and flow rate is 50 ml min^{-1}.

Charge/discharge process:

Anode: $Ni(OH)_2 + OH^- \rightarrow NiOOH + H_2O$

Cathode: $NiOOH + H_2O \rightarrow Ni(OH)_2 + OH^-$

Overcharge/discharge process:

Anode: $OH^- \rightarrow \frac{1}{2}O_2 + H_2O + 2e^-$

Cathode: $H_2O + 2e^- \rightarrow H_2 + 2OH^-$

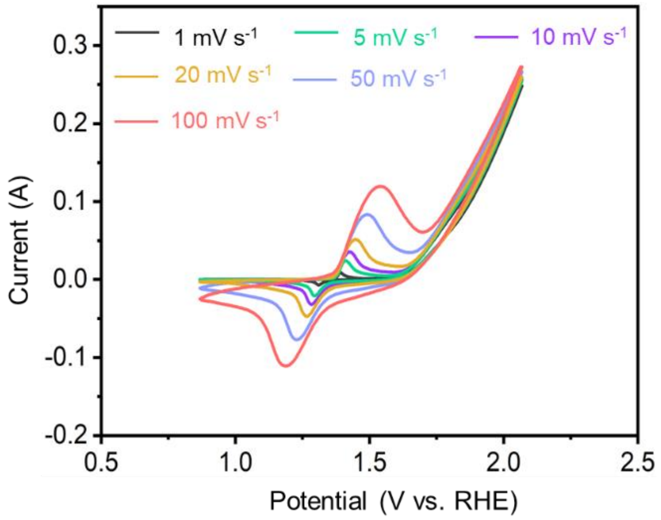

Supplementary Figure 7: CVs of a activated $Ni(OH)_2$ electrode at various scan rates. The redox behavior of $NiOOH/Ni(OH)_2$ is visible.

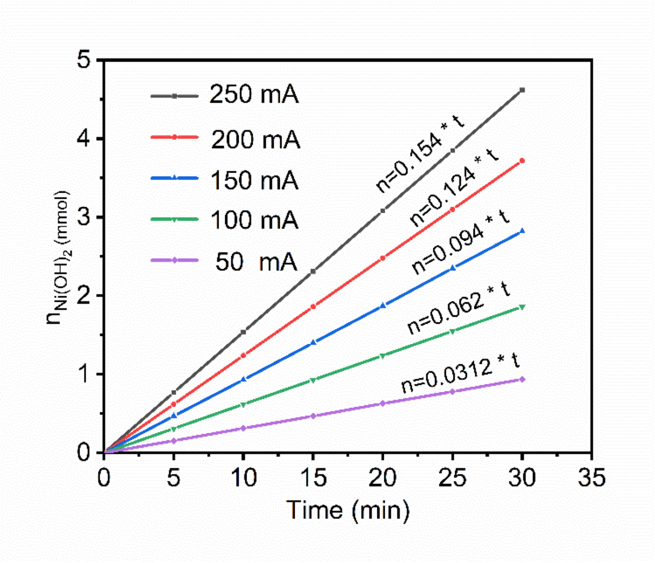

Supplementary Figure 8: The molar conversion of $Ni(OH)_2$ in MFE as a function of time at various currents.

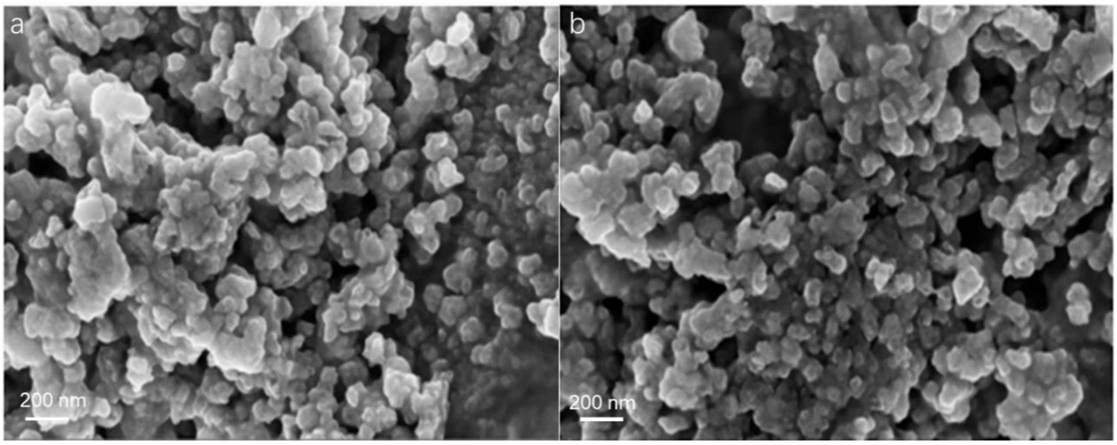

Supplementary Figure 9: SEM images of (a) Fe-Co bimetallic alloy/NC and (b) FeP-CoP/NC after phosphidation.

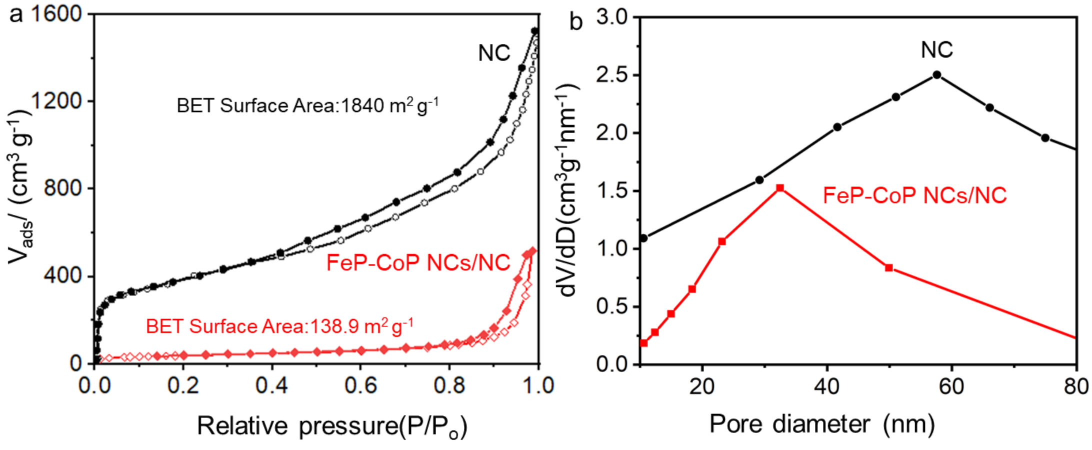

Supplementary Figure 10: The comparison of (a) N_2 adsorption isotherms and (b) pore size distribution of FeP-CoP/NC and NC.

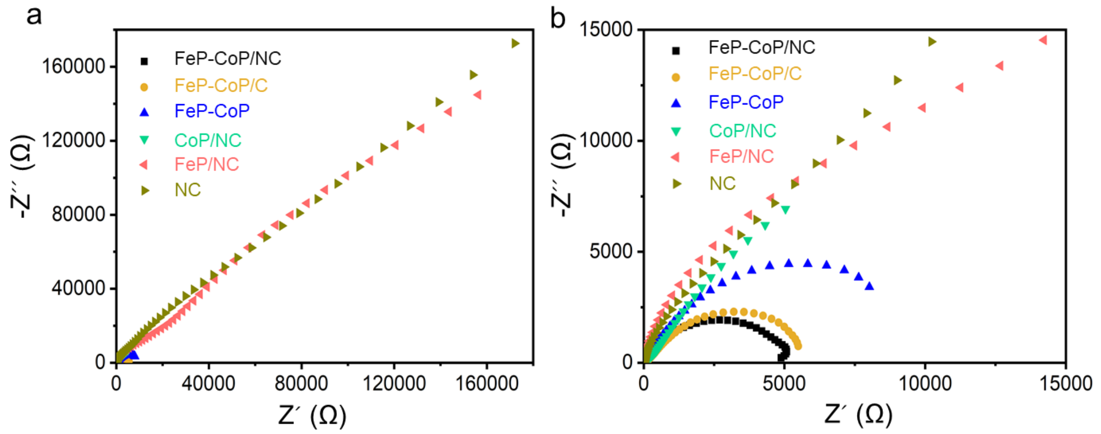

Supplementary Figure 11: EIS spectra of FeP-CoP/NC, FeP-CoP/C, FeP-CoP, CoP/NC, FeP /NC and NC in a 3-electrode system at open circuit condition. Electrolyte is N_2-saturated 1.0 M KOH.

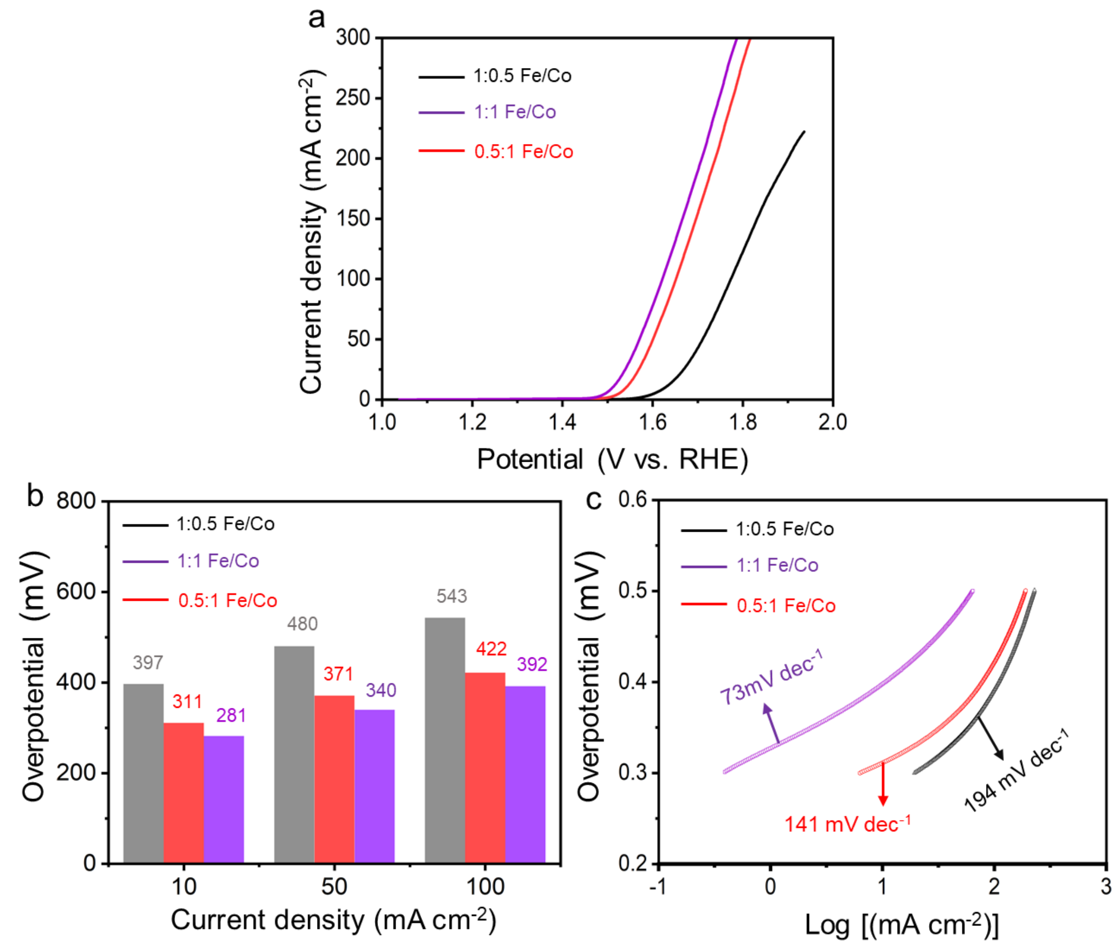

Supplementary Figure 12: Electrochemical performance of FeP-CoP/NC in OER with various Fe/Co ratio.

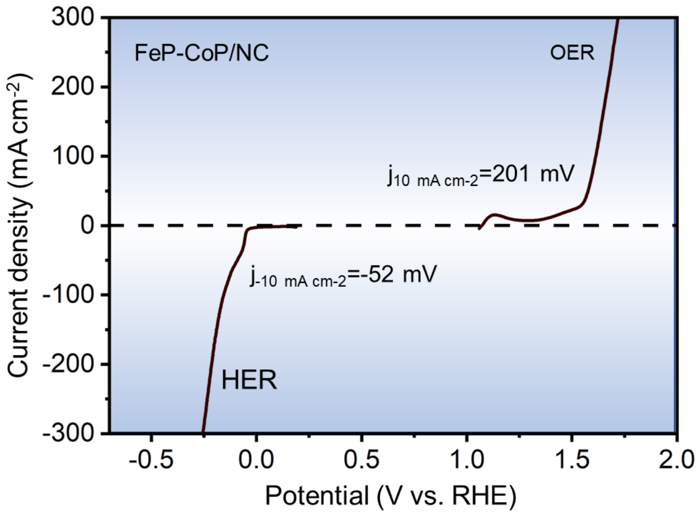

Supplementary Figure 13: The OER and HER performance of FeP-CoP/NC/CC in 1.0 M KOH solutions.

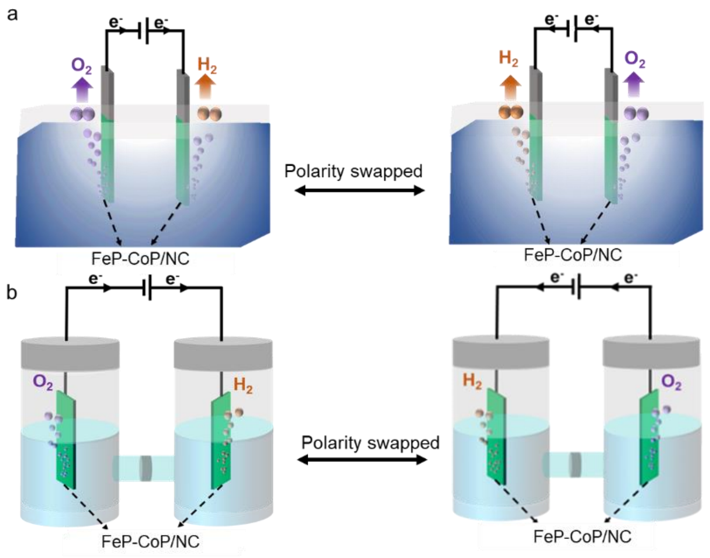

Supplementary Figure 14: The schematic assembly of (a) single-compartment cell and (b) H-cell for alkaline water splitting in this work.

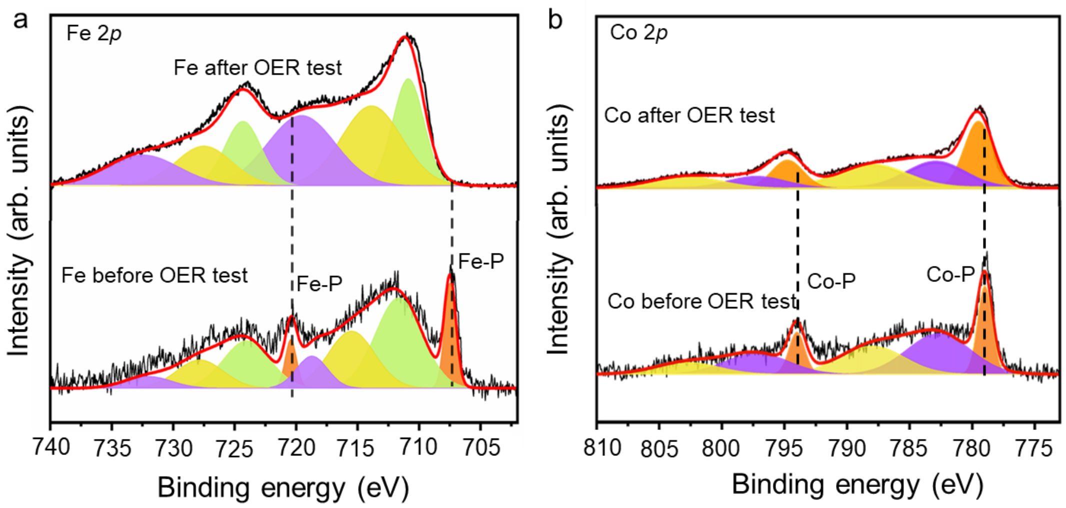

Supplementary Figure 15: high resolution XPS spectra of (a) Fe 2*p* and (b) Co 2*p* in FeP-CoP/NC before and after HER/OER cycle.

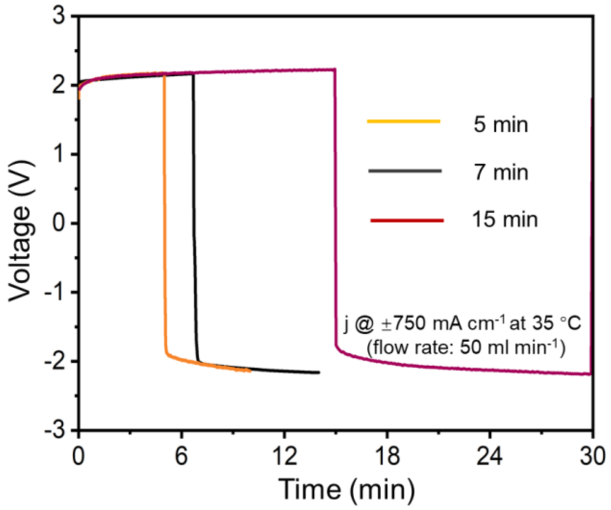

Supplementary Figure 16: Performance of MFE when the time per cycle is varied.

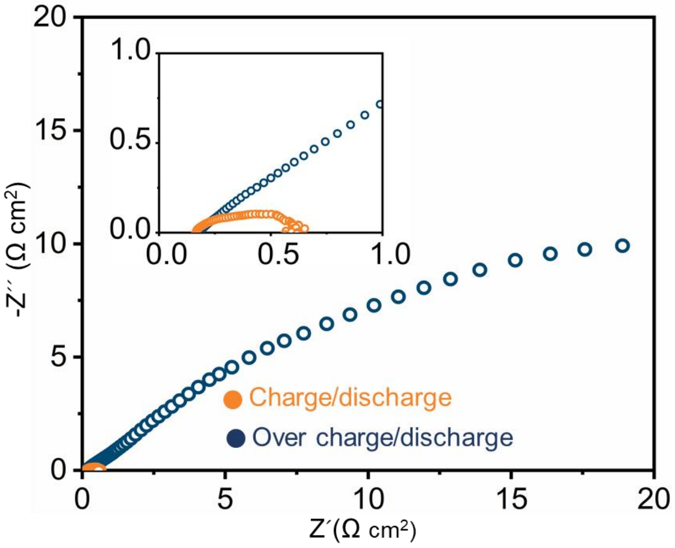

Supplementary Figure 17: The comparison of EIS spectra for a normal MFE and a MFE which is over (dis)charged.

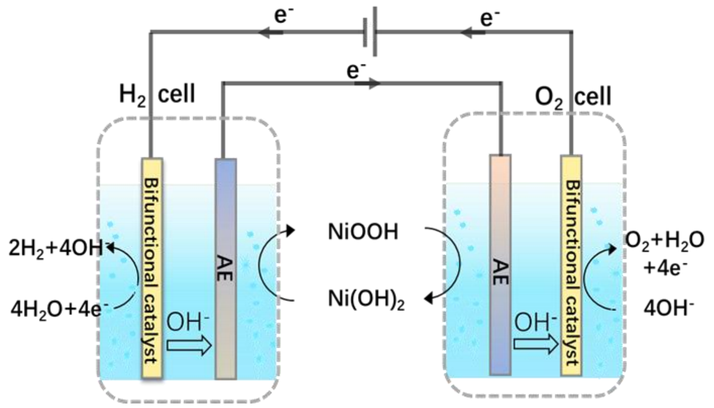

Supplementary Figure 18: The schematic of the electrolyzer using $NiOOH/Ni(OH)_2$ redox couple without flowing and MEA structure.

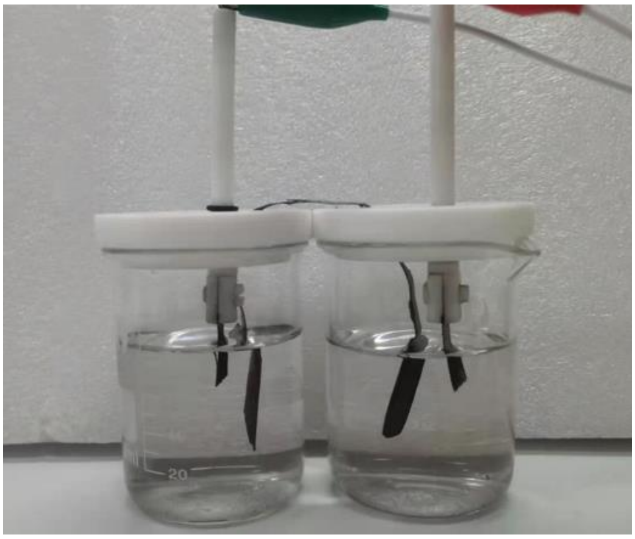

Supplementary Figure 19: Photograph of the conventional decoupled electrolyzer using $NiOOH/Ni(OH)_2$ redox couple.

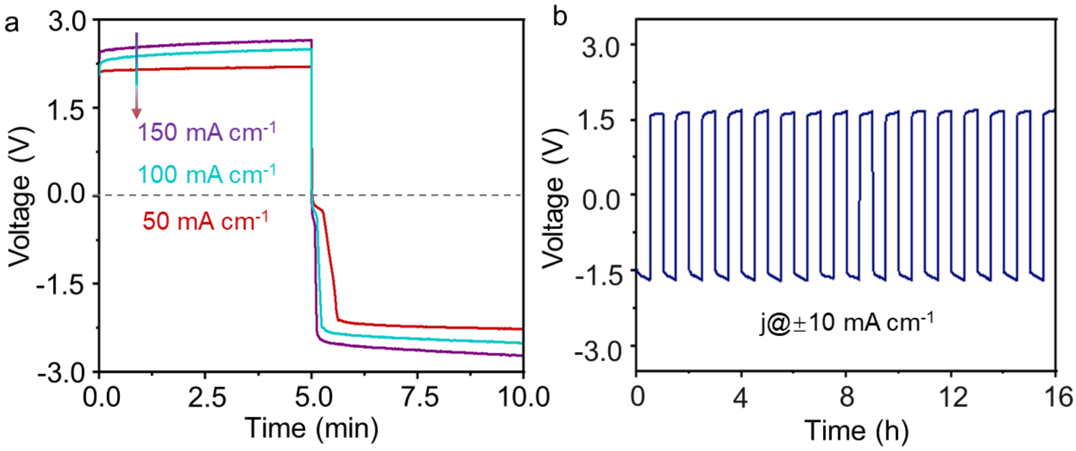

Supplementary Figure 20: (a) The *V*–*t* curve at ±10 mA cm^{-2}; (b) Cyclic stability test at ±10 mA cm^{-2} in conventional electrolyzer using redox couples. Temperature is 35 ^{o}C. Both anolyte and catholyte are 1.0 M KOH solutions. The cycle stability lowered by 10% from first cycle to the sixteen cycle.

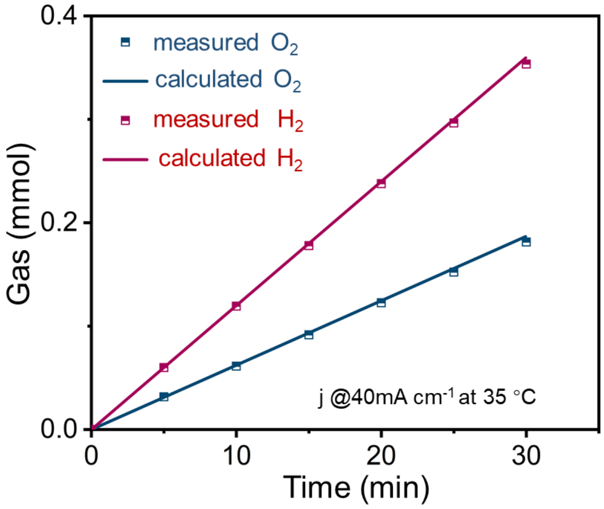

Supplementary Figure 21: Measured H_2 and O_2 yields in conventional electrolyzer using redox couples, solids lines represent the gas evolution at 100% Faradaic efficiency at current density of 40 mA cm^{-2}. Both anolyte and catholyte are 1.0 M KOH solutions. After 30 min, 0.353 mmol of H_2 has been measured, which was close to the theoretical value (0.356 mmol). The calculated Faradaic efficiency was 96.2%.

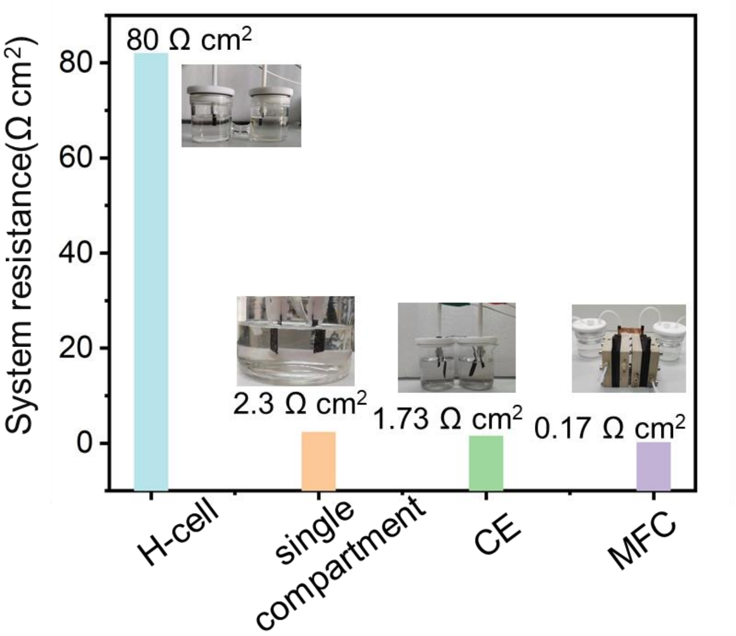

Supplementary Figure 22: The internal ohmic resistance of cells with different architectures (H-cell, single-compartment, electrolyzer using NiOOH/$Ni(OH)_2$ redox couple without flowing and sandwiched electrode structure), all electrodes are employed with FeP-CoP/NC catalysts in 1.0 M KOH solutions.

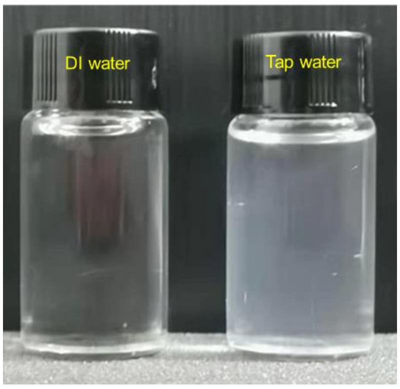

Supplementary Figure 23: The photograph of deionized water (left) and tap water electrolyte (right), Note the precipitation in the tap water electrolyte after adding KOH.

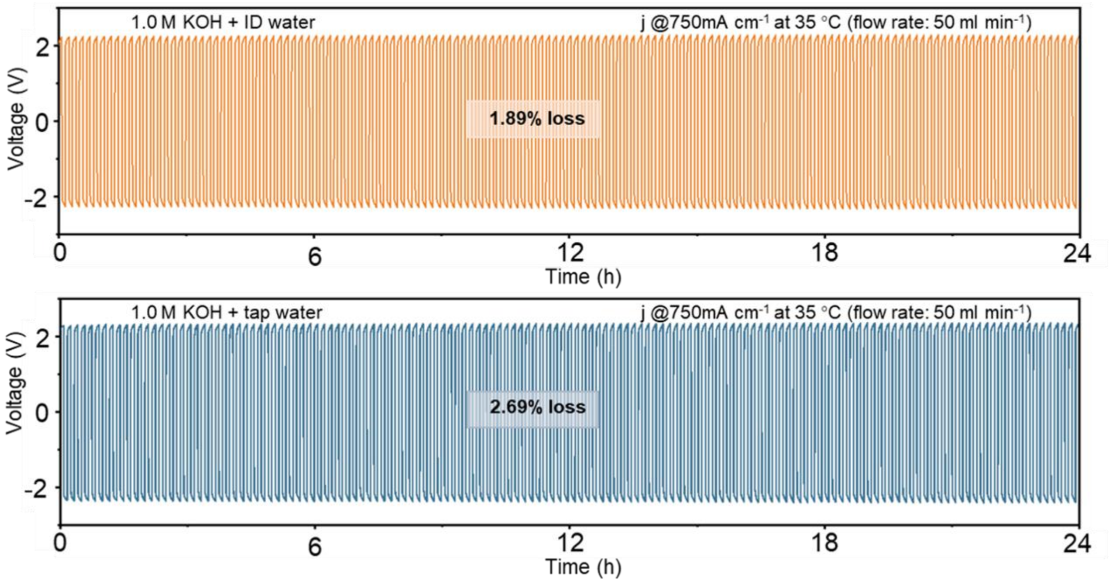

Supplementary Figure 24: Rescaled plots of the cyclic stability test of MFE in Figure 5c and 5d.

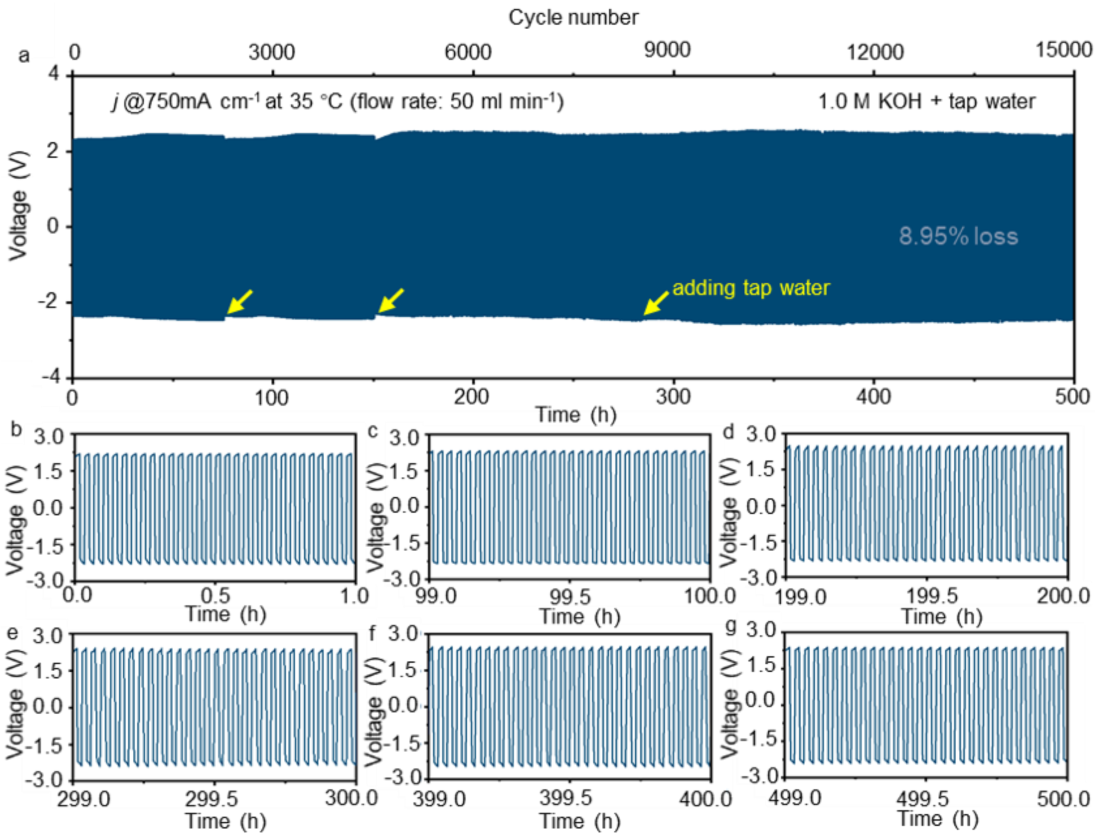

Supplementary Figure 25: A 500 h longevity test of MFE in tap water electrolyte. For the evaluation of the cyclic stability of the auxiliary electrode, 2 min per cycle was applied to achieve a higher cycle number.
